# The emerging role of transcriptional regulation in the oocyte-to-zygote transition

**DOI:** 10.1371/journal.pgen.1008602

**Published:** 2020-03-05

**Authors:** Paulo Navarro-Costa, Rui Gonçalo Martinho

**Affiliations:** 1 Instituto Gulbenkian de Ciência, Oeiras, Portugal; 2 Instituto de Saúde Ambiental, Faculdade de Medicina, Universidade de Lisboa, Lisboa, Portugal; 3 Center for Biomedical Research, Universidade do Algarve, Faro, Portugal; 4 Instituto de Medicina Molecular João Lobo Antunes, Faculdade de Medicina, Universidade de Lisboa, Lisboa, Portugal; 5 iBiMED, Departamento de Ciências Médicas, Universidade de Aveiro, Aveiro, Portugal; Geisel School of Medicine at Dartmouth, UNITED STATES

Fertilization marks the beginning of a new life by converting two terminally differentiated gametes into a single totipotent zygote. Central to this transition is a complex biological program commonly referred to as oocyte activation—an umbrella term for a series of profound changes that prepare the fertilized oocyte for totipotency [1, 2]. These include, among others, the completion of meiosis, the formation of the two pronuclei, and the selective translation of maternal RNAs. A remarkable aspect of oocyte activation is that it occurs in the absence of transcription. Not surprisingly, most of our knowledge of this process is centered on the posttranscriptional regulation of gene expression [3]. Yet, a recent body of evidence has brought new focus on the fundamental importance of transcriptional regulation during oogenesis as a primer for the oocyte-to-zygote transition [4].

In this issue of *PLOS Genetics*, Torres-Campana and colleagues [5] provide new compelling data further supporting the view that developing female germ cells rely on highly specific gene expression programs to later sustain oocyte activation and the oocyte-to-zygote transition. Through a female germ line-specific RNA interference screen in *Drosophila melanogaster*, the authors identified distinct chromatin remodelers required for the assembly of the paternal pronucleus at fertilization: lysine-specific demethylase 5 [Kdm5; also known in *Drosophila* as little imaginal discs (Lid)] and its interacting partners, Sin3A and histone deacetylase 1 (HDAC1/Rpd3). In the absence of these transcriptional regulators, they observed that fertilized oocytes failed to efficiently remove protamines from sperm chromatin—an essential step for the subsequent merging of the two parental chromosome sets in early embryogenesis. Importantly, Kdm5 was also required for migration of the female pronucleus, clearly suggesting multiple functional requirements of this demethylase for successful karyogamy.

The KDM5 family of proteins are Jumonji C lysine demethylases that remove methyl groups from tri-methylated and di-methylated lysine 4 on histone H3 (H3K4) [6]. Although tri-methylation of H3K4 (H3K4me3) is unlikely to be a primary regulator of gene expression [7, 8], this histone mark is highly enriched at the transcription start site of active genes [9] and may be an important aspect of gene expression robustness. Yet, the recruitment of Kdm5 to promotors is just one of the mechanisms through which these proteins can regulate transcription [10, 11]. In fact, Kdm5 can also regulate gene expression independently of its demethylase activity, through its interaction with lysine deacetylases (HDACs) and the nucleosome remodeling and deacetylase (NuRD) complex [12, 13]. Therefore, Kdm5 can behave, depending on the cellular context, as a positive or negative regulator of gene expression.

Kdm5 demethylases are crucial for the coordination of gene expression programs across different developmental contexts [14]. More recently, their important functions during oogenesis have begun to be uncovered. We and others have shown that *Drosophila* Kdm5 restricts the levels of H3K4me3 in developing female germ cells and is required both for synaptonemal complex assembly and the temporal control of gene expression during meiosis [15, 16]. Such functions highlight the role of these enzymes as potent chromatin remodelers during female gametogenesis, which is further illustrated by the significant defects in meiotic and postmeiotic chromosome architecture observed in Kdm5-depleted oocytes [15, 16]. Not surprisingly, the loss of Kdm5 and its demethylase activity are associated with a significant reduction of female fertility [15].

Now Torres-Campana and colleagues [5] further expand the gamut of Kdm5-related functions during oogenesis to include the transcriptional regulation of deadhead (dhd)—a major player in the oocyte-to-zygote transition. dhd encodes a thioredoxin-like protein that was previously described to be essential for sperm chromatin decompaction after fertilization [17]. The authors observed that dhd expression was severely reduced in Kdm5-depleted oocytes [5], which is in accordance with their recorded incapability of forming the paternal pronucleus. Importantly, Kdm5 depletion was associated with a sharp decrease of H3K4me3 across the dhd gene body, suggesting that Kdm5 directly regulates dhd expression. Although this counterintuitive loss of H3K4me3 after depletion of a histone demethylase would favor a demethylase-independent function of Kdm5, this does not seem to be the case. In fact, in a rather surprising twist of events, a demethylase-inactive allele of Kdm5 failed to rescue dhd expression levels in Kdm5-depleted oocytes.

How can we reconcile the seemingly contradictory observations that Kdm5 demethylase activity is required for dhd expression but the absence of Kdm5 is in itself associated with low levels of H3K4me3 in dhd? Two nonmutually exclusive hypotheses can be envisaged. In the first, Kdm5 regulates H3K4me3 levels in a yet unknown enhancer sequence responsible for the control of dhd expression. In the second, a specific Kdm5-mediated organization of germ-cell chromatin architecture may be required for the efficient expression of dhd. Under this hypothesis, we can posit a scenario where Kdm5 depletion impairs the organization of topologically associating domains (TADs) due to increased H3K4me3 and/or reduced activity of Kdm5-interacting partners (such as the SIN3 histone deacetylase complex).

An important point from Torres-Campana and colleagues’ study [5] regards the experiment in which the authors attempt to suppress, via ectopic expression of dhd, the sperm chromatin-remodeling defects of Kdm5-depleted oocytes. The outcome of this experiment was a quite modest degree of functional rescue, which clearly suggests that besides dhd, Kdm5 regulates the expression of other genes similarly important for postfertilization development. Such observation parallels our previous results on the mixed-lineage leukemia 3/4 (MLL3/4) histone methyltransferase [known in *Drosophila* as dMLL3/4 or trithorax-related (Trr)], in which we could demonstrate that the activity of this chromatin remodeler during oogenesis promoted the expression of a subset of genes later required for different aspects of the oocyte-to-zygote transition [4]. Quite significantly, the loss of this methyltransferase was associated with a broad palette of oocyte activation defects, such as the incapability of completing meiosis, forming the paternal pronucleus, and initiating the embryonic mitotic divisions.

**Fig 1:**
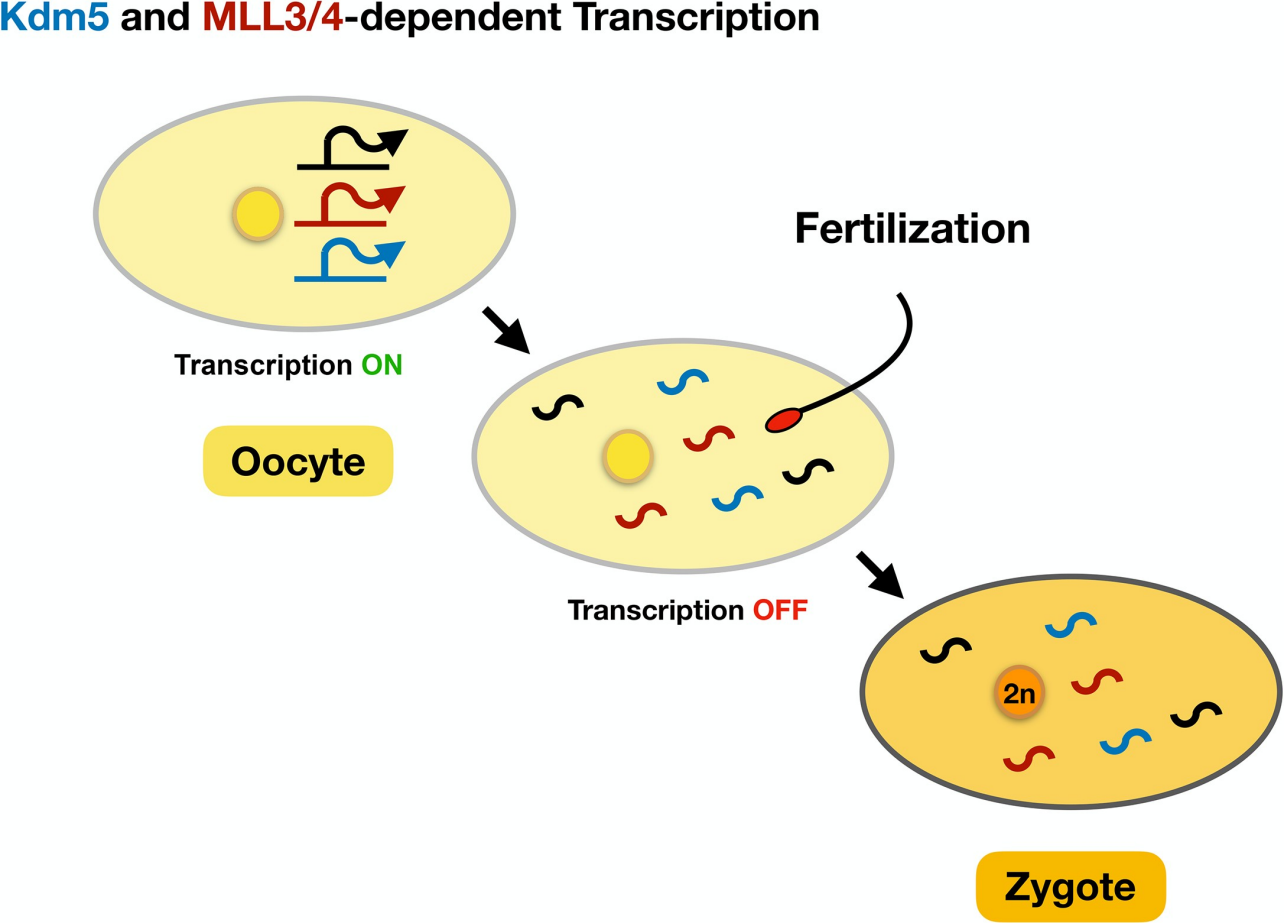
The emerging role of transcriptional regulation in the oocyte-to-zygote transition. Female germ cells rely on Kdm5 and on the MLL3/4 histone methyltransferase to regulate highly specific gene expression programs that are later required for the oocyte-to-zygote transition. Kdm5, lysine-specific demethylase 5; MLL3/4, mixed-lineage leukemia 3/4.

Collectively, these studies emphasize the point that transcriptional regulation during oogenesis prepares the oocyte for activation (Fig 1). We can thus hypothesize that different epigenetically defined gene expression modules establish, as the oocyte develops, the molecular basis of the future oocyte-to-zygote transition. In this regard, it is important to mention that the disruption of histone demethylases during mouse oogenesis [including lysine-specific demethylase 5B (Kdm5b)] has also been associated with meiotic defects and female infertility [18]. It is therefore likely that some of the key molecular players responsible for orchestrating oocyte maturation and the oocyte-to-zygote transition are evolutionarily conserved between *Drosophila* and mammals. Accordingly, exploring the role of chromatin-remodeling enzymes such as Kdm5 in the context of human fertility represents an exciting and still clinically underexplored topic.
